# Diagnostic and prognostic value of cardiovascular magnetic resonance in non-ischaemic cardiomyopathies

**DOI:** 10.1186/1532-429X-14-54

**Published:** 2012-08-02

**Authors:** Chirine Parsai, Rory O’Hanlon, Sanjay K Prasad, Raad H Mohiaddin

**Affiliations:** 1 Cardiovascular Magnetic Resonance Unit, Royal Brompton and Harefield NHS Trust, London, UK; 2 Cardiology and CMR Unit, Polyclinique Les Fleurs, Toulon, France; 3 Centre for Cardiovascular Magnetic Resonance, Blackrock Clinic, Dublin, Ireland; 4 National Heart and Lung Institute, Imperial College, London, UK

## Abstract

Cardiovascular Magnetic Resonance (CMR) is recognised as a valuable clinical tool which in a single scan setting can assess ventricular volumes and function, myocardial fibrosis, iron loading, flow quantification, tissue characterisation and myocardial perfusion imaging.

The advent of CMR using extrinsic and intrinsic contrast-enhanced protocols for tissue characterisation have dramatically changed the non-invasive work-up of patients with suspected or known cardiomyopathy.

Although the technique initially focused on the in vivo identification of myocardial necrosis through the late gadolinium enhancement (LGE) technique, recent work highlighted the ability of CMR to provide more detailed in vivo tissue characterisation to help establish a differential diagnosis of the underlying aetiology, to exclude an ischaemic substrate and to provide important prognostic markers.

The potential application of CMR in the clinical approach of a patient with suspected non-ischaemic cardiomyopathy is discussed in this review.

## Review

### Introduction

Cardiomyopathies encompass a broad spectrum of myocardial conditions which can affect the heart as a primary disease process or as part of a systemic disorder, evolving toward heart failure or cardiovascular death.

Progress of modern molecular biology and its introduction into clinical cardiovascular medicine has considerably changed the approach to cardiomyopathies and has led to new classification schemes. .While cardiomyopathies were initially defined as disorders that were idiopathic, expert panels classify cardiomyopathies now into: primary, acquired and mixed [1]. This results from the data published by the latest AHA (2006) and ESC (2008) classifications segregating cardiomyopathies into familial/genetic and non-familial/non-genetic [2,3]. Both recommendations define a cardiomyopathy as ‘a myocardial disorder in which the heart muscle is structurally and functionally abnormal in the absence of coronary heart disease, hypertension, valvular heart disease and congenital heart disease sufficient to cause the observed myocardial abnormality’ [3]. Cardiomyopathies are therefore grouped into specific morphological and functional phenotypes and sub-classified into familial and non-familial forms.

Whilst prevalence of dilated cardiomyopathy reaches 36 cases per 100 000 population, these figures as well as those reported for other cardiomyopathies, underestimate the frequency of the disorder as a number of patients are asymptomatic until reaching advanced stages of the disease. In addition, accurate diagnostic and prognostic phenotyping of cardiomyopathy remains a challenge owing to frequent overlapping features between individual cardiomyopathies.

In the investigative workup for a suspected cardiomyopathy, typically a considerable number of tests are performed, ranging from the more routine and straightforward tests including ECG, echocardiogram, and exercise treadmill, to the more invasive including coronary angiography, electrophysiological studies, and endomyocardial biopsy. It is key to identify a potentially treatable substrate and then to risk stratify patients for treatment including consideration of an implantable defibrillator (ICD) or cardiac resynchronisation device.

Advances in cardiovascular magnetic resonance (CMR) provide the potential to address all these important issues in a single scan setting complementing other non-invasive tools and genetic testing. The cost-effectiveness of non-stress CMR has been highlighted recently pointing out the ability of CMR to act as a cost-reducing gatekeeper to invasive coronary angiography in specific clinical settings [4].

In a single 45–60 minute study, CMR can provide 3-dimensional data on cardiac anatomy, function, tissue characterisation, coronary and microvascular perfusion, valvular disease in any selected plane, regardless of patient’s habitus and without ionising radiation. The relative safety of gadolinium agents and tissue characterising sequences allows for repeated imaging, follow-up, family screening and serial risk stratification.

In this review, non-ischaemic cardiomyopathies (NICMP) are approached from a clinical point of view outlining the diagnostic and prognostic role of CMR as an integral part of the clinical decision making algorithm.

## Technical aspects

**Figure 1:**
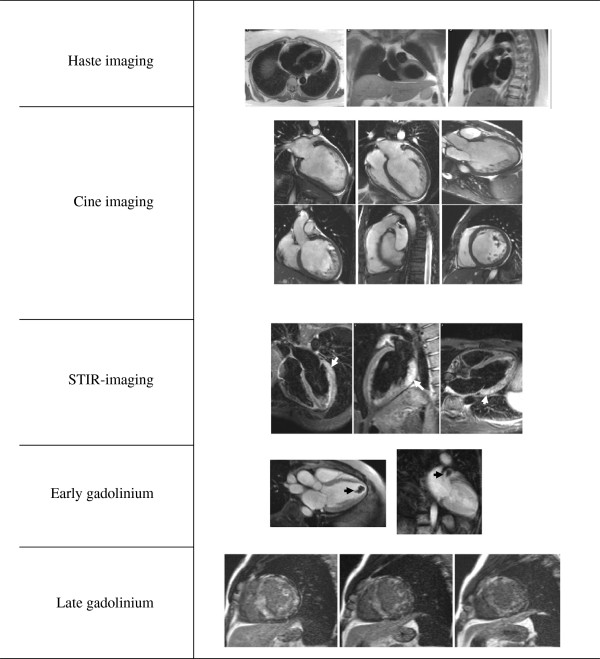
**Standard cardiomyopathy protocol.** Standard cardiomyopathy protocol including HASTE, cine imaging, T2-weighted-imaging (STIR) searching for inflammation/edema (white arrows), early gadolinium imaging to detect thrombus (black arrow) and LGE to identify fibrosis or infiltration (diffuse LGE typical of amyloidosis).

In the imaging workup of a suspected cardiomyopathy a wide range of sequences are acquired following dedicated protocols [5-8]. Figure 1 summarises a typical cardiomyopathy protocol which will be modulated according to the clinical suspicion of the underlying disease and throughout the scan on the basis of the evolving picture. Table 1 provides an overview of the technical aspects of the main sequences and Table 2 highlights strengths and weaknesses for each of them.

**Table 1:** Technical characteristics of standard CMR sequences

**Sequence**	**Pixel size (mm) Read FOV 340 mm**	**Pixel size (mm) Phase FOV 340 mm**	**Flip angle (degrees)**	**TE (ms)**	**TR (ms)**	**TI (ms)**	**Interslice gap (mm)**	**Slice thickness (mm)**	**Raw data (lines/cycle or turbo factor)**
**Breath-hold balanced SSFP cine**	1.8	1.8	80	1.3	40(2.6)		3	7	15
**T2W-TSE with dark blood preparation and fat-suppression by STIR (short tau inversion recovery)**	1.3	2.3		47	Alternative cardiac cycles	170	3	7	15
**Early Gd enhancement (inversion-recovery spoiled gradient-echo)**	1.3	1.9	40	1	Alternative cardiac cycles	400	3	7	25
**Late Gd enhancement (inversion-recovery spoiled gradient-echo)**	1.3	1.9	40	1	Alternative cardiac cycles	260-400	3	7	25

**Table 2:** Strengths and weaknesses of standard CMR sequences

**Sequence**	**Purpose**	**Strengths**	**Weaknesses**
**Breath hold balanced SSFP cine**	Global regional myocardial function and wall thickness	High SNR (T2/T1) image contrast gives reliable blood: myocardium endocardial border definition based on long T2 of blood	Sensitive to field inhomogeneity (off- resonance banding artefact). Unreliable appearance. ECG-mistriggering/Poor breath hold
**T2W-TSE with dark blood preparation and fat-suppression by STIR(short tau inversion recovery)**	Oedema, Infiltration	Detection of myocardial fluid content by longer T2 relaxation time	Low SNR
			Incomplete blood suppression at endocardial boundary layer
			Unreliability due to diastolic cardiac motion ECG mistriggering
			Poor breathhold
**Stress first-pass myocardial perfusion**	Cardiomyopathy related perfusion defects/exclusion of concurrent epicardial coronary artery disease	Higher resolution than SPECT	Incomplete myocardial coverage
			“Dark-rim” artefact Cardiac motion during image
**Early Gd enhancement (inversion-recovery spoiled gradient-echo**	Microvascular obstruction Detection of intracardiac thrombus	High spatial resolution (compared to CMR perfusion)	Dependent on image timing after injection. ECG mistriggering
			Poor breath hold
**Late Gd enhancement (inversion recovery spoiled gradient-echo)**	Myocardial fibrosis	High T1 contrast of diseased myocardium	Image contrast depends on inversion time adjustment and Gad washout. Ghosting from long T1 fluids (can be suppressed).
			ECGmistriggering
			Poor breath-hold
**Real-time or single-shot versions of many sequences above**	As above	No breath-holding Tolerates ECG mistriggering	Coarser image resolution.
			Cardiac motion during image.

## Gross anatomical assessment

Dark-blood imaging using mutislice single-shot spin-echo sequence (Half-Fourier Acquisition of Single-Shot Turbo Spin-Echo, HASTE) in trans-axial, coronal and sagittal planes allows a gross anatomical assessment in under 3 min. Alternatively 10–12 s breathhold images in each of the imaging planes using bright-blood imaging with steady-state free precession sequence (SSFP) can be performed.

## Dynamic imaging

Dynamic long-axis and short-axis views of the heart are then acquired using breath-hold cine-SSFP imaging. Cine-CMR is accepted as the gold standard for accurate and reproducible quantification of left and right ventricular volumes, biventricular ejection fraction, and left ventricular (LV) mass obtained through manual planimetry or the use of semi-automated software [9]. Presence of normalised values for CMR dimensions corrected for age, sex and body surface area helps to diagnose subtle ventricular dysfunction with greater accuracy [10]. However, motion or breathing artifacts in sicker patients and irregular rhythms (atrial fibrillation or frequent ectopics) add to the complexity of acquiring high quality cine images, and in some cases real-time cine imaging is performed with an acceptable reduction in resolution providing analysis of ejection fraction (EF), volumes, and mass closely correlating with values obtained using echocardiography and conventional magnetic resonance imaging [11-13].

## Non-contrast Tissue characterisation

Specialised sequences allow characterisation of pathological tissue without the need for a contrast agent. The T2-weighted sequence, Short-Tau Inversion Recovery (STIR) is a powerful sequence for imaging oedema suppressing signal from flowing blood and from fat enhancing sensitivity to myocardial fluid content. Long T2 relaxation times of water-bound protons are used to generate a water-specific contrast resulting in high signal intensity of oedematous tissue [14,15]. STIR imaging can therefore detect acute myocyte swelling and interstitial fluid accumulation [16]. Initially validated in the ischaemic model, its use has been extended to NICMP, particularly myocarditis, stress cardiomyopathy and transplant rejection [17-20].

Pitfalls include low signal-to-noise ratio, surface coil intensity variation, interfering bright signal from stagnant blood, motion artifacts and the subjective nature of interpretation [21,22]. Some of these issues can be addressed using direct quantification of T2 by mapping techniques or shorter acquisition protocols with single-shot techniques [23,24]. Hybrid sequences combining turbo spin echo and SSFP have been proposed to eliminate imperfect blood suppression [2].

T1-weighted and turbo-spin echo sequences are useful to assess the pericardium and to look for fat infiltration.

Myocardial T2*-mapping is a unique technique, validated against in-vivo histology, allowing direct identification and quantification of myocardial iron in vivo [25].

## Imaging Fibrosis or Infiltration

Myocardial fibrosis or infiltration can be assessed following administration of gadolinium. Gadolinium is an extracellular agent accumulating in areas of interstitial expansion (due to myocardial fibrosis, oedema or infiltration). Three phases can be assessed after gadolinium injection: the first pass (immediate imaging at rest or during stress), early enhancement (first 5 min) and late enhancement (5 to 20 min after injection). First pass can be used for perfusion imaging and is often given during vasodilator stress to detect myocardial ischaemia. Following intravenous injection of gadolinium (0.1-0.2 mmol/kg body weight), the contrast media is followed through the LV at rest and during pharmacologic stress to assess homogeneity of perfusion [26,27]. Ultra-fast acquisition is essential and protocols are usually based on gradient-echo or SSFP sequences combined with parallel imaging acquiring three to five slices (short-axis and/or long-axis) [28]. First-pass Stress perfusion CMR can visualise inducible perfusion defects occurring at the microvascular level in NICMP as circumferential areas of subendocardial hypoperfusion resolving on resting images.

Early gadoliuium enhancement (EGE) imaging (1–3 min after injection) enables the detection of hypovascular regions such as dark, unenhanced intra-cardiac thrombi, and microvascular obstruction (post myocardial infarction).

Late gadolinium enhancement (LGE) imaging (5–20 min after injection) detects accumulation of contrast in areas of infarction or fibrosis due to slower contrast kinetics and greater volume of distribution in extracellular matrix.

This is performed using inversion recovery Turbo-FLASH gradient echo or phase-sensitive inversion recovery (PSIR) in identical planes to the cine images searching for areas of fibrosis or infiltration. PSIR provides corrected images that are virtually insensitive for pre-pulse delay timing. It allows for a faster acquisition method without adjustment of inversion time and more consistent image quality to the expense of a lower spatial resolution [29,30]. This is of particular interest in patients having difficulties performing breath-holds or in presence of arrhythmias. PSIR appears as a reliable technique more sensitive for detection of small, diffuse or sub-endocardial lesions due to a better contrast when compared to a standard 2-dimension (2D) or 3-dimension (3D) inversion recovery gradient echo sequence [31,32].

**Figure 2:**
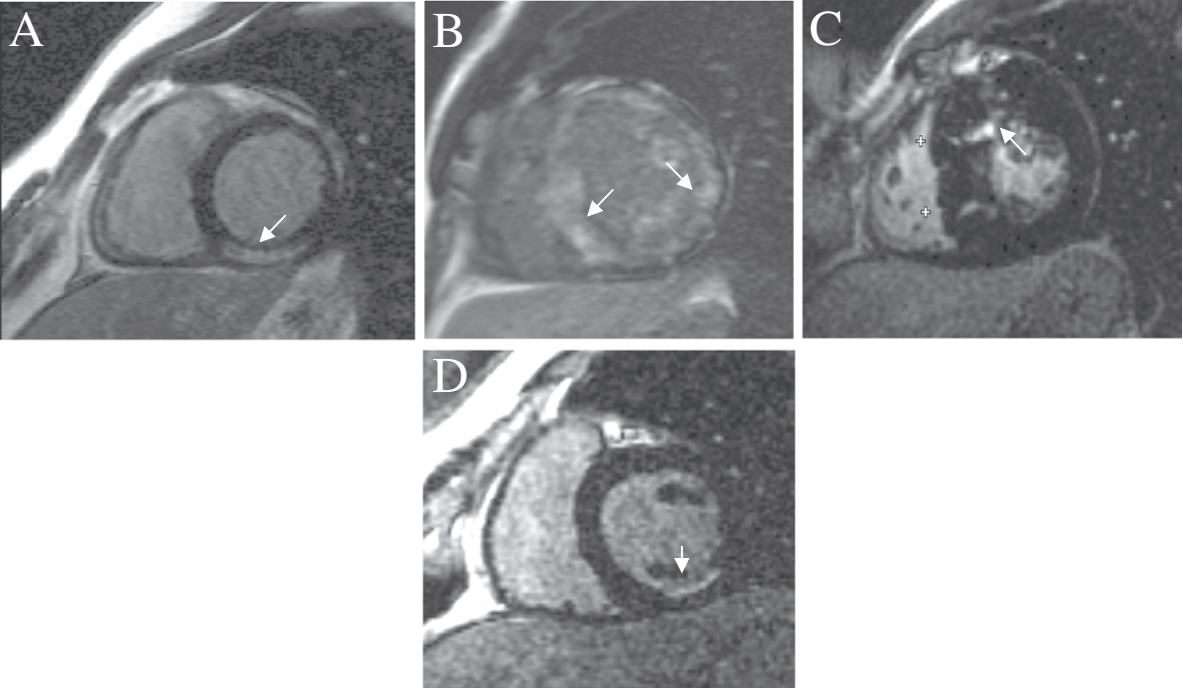
**Diagnostic patterns of LGE.** Distribution pattern and location of LGE contributes to the differential diagnosis between a NICMP (**A** : sub-epicardial fibrosis following myocarditis, **B** : circumferential diffuse enhancement in amyloidosis, **C** : patchy fibrosis in affected hypertrophied segments in HCM) and a typical ischaemic sub-endocardial enhancement (**D**).

Using inversion recovery sequences, focal fibrosis is imaged as a localised area of high signal intensity. The extent and pattern of LGE varies according to the underlying pathological process and contributes therefore to establish the correct diagnosis in NICMP (Figure 2).

Presence of fibrosis in NICMP is often only identified visually but its extent can be quantified as a percentage of total LV mass using dedicated software [33]. All quantification techniques rely on the fact that LGE makes the scar appear bright and as such can be defined as a signal intensity above normal myocardium with 2 standard deviations (SDs) being advocated by official guidelines. Other techniques are also used with 3, 4, 5 or 6 SDs, manual quantification and the full-width half maximum technique (FWHM) using half of the maximal signal within the scar as the threshold. While the officially recommended 2-SD technique can double the LGE volume compared with manual, FWHM, and 5 or 6-SD techniques, the FWHM technique appeared as the most reproducible method for quantification of fibrosis regardless of etiology (fibrosis due to myocardial infarct or HCM) and compared to ex vivo data [33-35]. However, CMR spatial resolution limits evaluation of microscopic interstitial fibrosis and LGE correlates poorly with collagen volume calculated from endomyocardial biopsies [36]. A promising approach to overcome this limitation relies on the use of T1 mapping [37].

### T1 mapping

LGE imaging sequences delineate fibrosis by revealing a relative difference in T1-relaxation times between areas of scar (T1 shortened by accumulation of gadolinium) and normal myocardium (T1 closer to normal as gadolinium is rapidly washed out). T1-mapping techniques work by measuring the absolute T1-relaxation time for all areas of myocardium on a pixel by pixel basis. As the shortening of T1 relaxation time is proportional to the local concentration of gadolinium, this can reveal subtle changes in T1 times due to expansion of the interstitial space with collagen and other fibrous tissue components. Early T1 mapping techniques were very time consuming. A Look-Locker sequence consisting of a gradient echo cine sequence with a nonslice selective inversion pulse after an R wave followed by a segmented gradient echo acquisition was developed [38,39]. Mean signal intensities obtained both before and at least twice after contrast administration, were plotted against the delay after the inversion pulse, deriving T1 values and allowing calculation of a myocardial gadolinium contrast partition coefficient. The MOdified Look-Locker Inversion recovery pulse sequence (MOLLI sequence) is a popular approach allowing measurement of T1 times in a single breath hold [40,41]. A Shortened MOLLI (ShMOLLI) sequence has also been recently tested, using sequential inversion recovery measurements within a single shortened breath hold [42,43].

The local concentration of gadolinium will be affected by several factors including the amount of scar, the rate at which gadolinium is cleared from the body but also by the amount of extracellular fluid available in the body for the contrast to distribute into [44]. With the knowledge of the patient’s haematocrit, simple kinetic models allow these corrections to be made and a standardised estimate of the extracellular volume fraction, V_e_, (an index of fibrosis if the extracellular space is occupied by scar tissue) to be determined [39,44].

While T1 mapping can accurately differentiate both interstitial and replacement fibrosis from normal myocardium, it cannot distinguish one type of fibrosis from another [45]. To date very few studies using T1 mapping in the clinical setting are published, but it is hoped that T1 mapping may provide quantitative assessment of diffuse myocardial fibrosis in patients with cardiomyopathies.

## Flow quantification

Despite a lower temporal resolution compared to echocardiography, assessment of valvular disease is achievable using CMR velocity mapping with reproducible results [46].

## Deformation imaging

**Figure 3:**
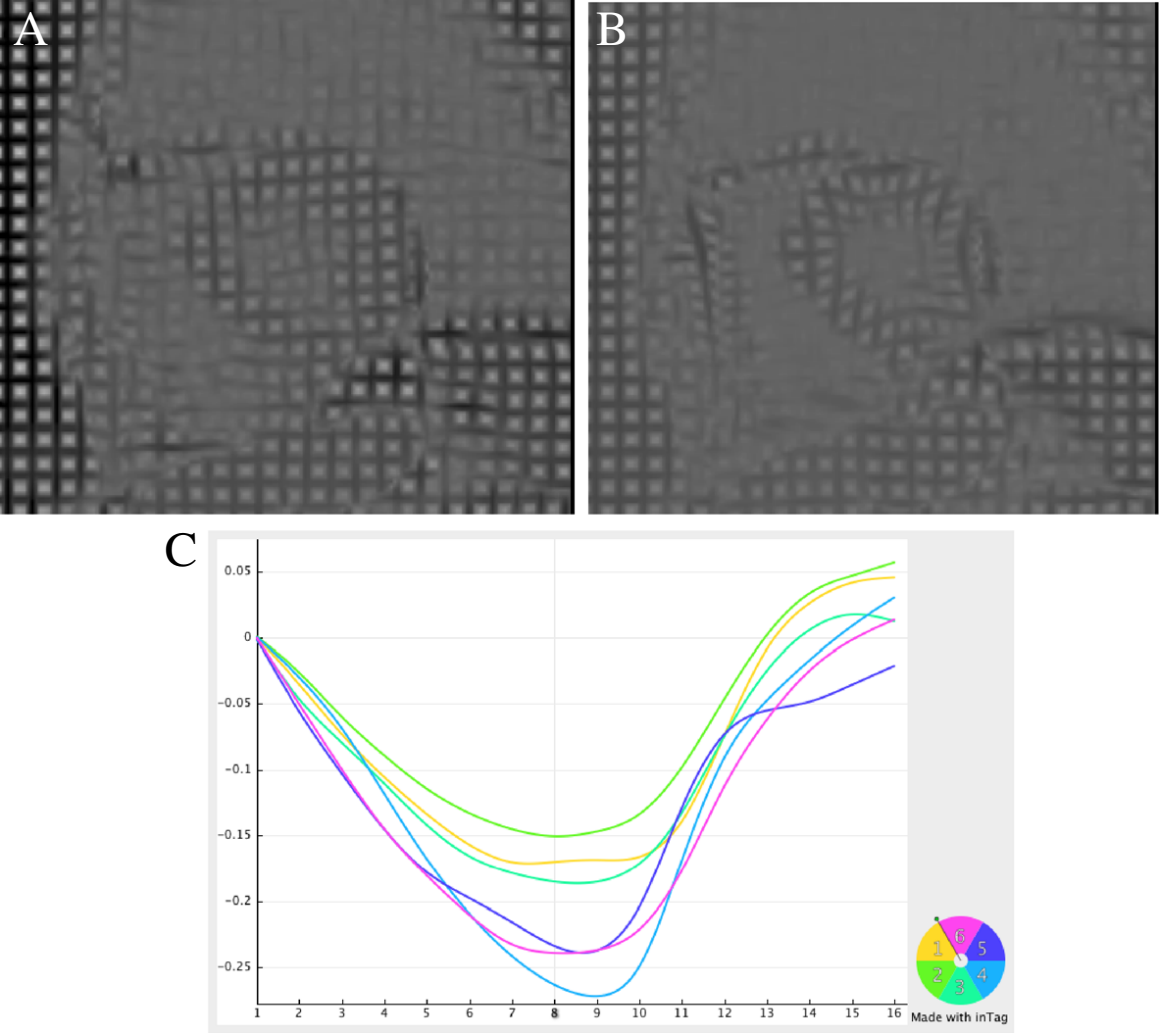
**Example of myocardial tagging using CSPAMM in an HCM patient (A: end diastole, B. end systole).** Deformation curves are extracted using a dedicated software (**C**). Courtesy of Dr Tevfik Ismael. Images were analysed with inTag (software supplied as part of a collaboration agreement with University of Lyon, Pierre Croisille and Patrick Clarysse, Creatis).

CMR myocardial tagging quantifies myocardial deformation, contraction and relaxation in the radial, circumferential and longitudinal directions adding useful information particularly prior to resynchronisation therapy (Figure 3). This technique and its applications have been recently reviewed [47].

A feature tracking method has recently been made available for the post processing of routinely acquired SSFP cines using inhomogeneities of the tissue as identifiers, which are then identified by software algorithms for analysing regional function. Following manual contouring of the LV endocardial border of the first cine frame, the software automatically identifies and tracks endocardial and epicardial myocardial boundaries through the remaining phases of the cardiac cycle. Although the method has been compared to echocardiographic speckle tracking, it may be better described as ‘boundary’ tracking when applied to CMR SSFP acquisitions. Potentially derivable parameters include circumferential, longitudinal and radially directed velocity, displacement, strain and strain rate. However, some of these derived measures even more than others require cautious interpretation with respect to both spatial and temporal resolution of cine images, and the design of the software. Feature tracking based measures of global strain of short axis cine slices – essentially the relative change of length of the tracked endocardial boundary – have shown correlation with corresponding global strain measured by CMR myocardial tagging, with reduced values found in patients with Duchenne muscular dystrophy compared with volunteers and appeared as a promising technique to detect wall motion abnormalities during Dobutamine stress CMR [48-50]. However, studies of reproducibility and accuracy of this potentially appealing analysis method have yet to be published, particularly for analyses of regional strain.

## Limitations

Amongst limitations of CMR, metallic implants and intracardiac Devices (pacemaker, ICD) represent the only absolute contra-indication, but this will be overcome in the near future by new generation of CMR compatible pacing leads currently in clinical trials. Severe claustrophobia, clinical instability and the first trimester of pregnancy are among relative contra-indications. Even though gadolinium contrast agents are relatively safe, their use is restricted in chronic renal failure (GFR <30 ml/min) due to the small but tangible risk of nephrogenic systemic fibrosis (NSF) which is enhanced by concomitant heart failure [51]. On the basis of current available data, in patients with severe renal failure, the first step is to determine whether a non-gadolinium sequence is available and could provide adequate data. If the clinical benefits of LGE-CMR outweighs the risk of NSF and if no other imaging method can be used to answer the clinical question, following a joint discussion between the referring physician and the patient, stable agents such as gadobenate dimeglumine should be used as the lowest possible dose, avoiding repeat scans within a short time period [52]. Amongst limitations for the use of CMR, the most restrictive issue probably still remains the limited distribution and availability of CMR scanners.

## Clinical aspects

Cardiomyopathy is often suspected on the basis of symptoms, an associated abnormal ECG and echocardiographic findings. Following detection of ventricular hypertrophy, dilatation (right or left ventricles) or abnormal wall motion abnormalities after acute chest pain syndromes with unobstructed coronaries, characteristic imaging features on CMR greatly assist in reaching a diagnosis and providing prognostic information. The diagnostic and prognostic abilities of CMR in these various clinical conditions are reviewed. Detection of a hypertrophied LV highlights the differential diagnosis between a physiologic response to pressure overload (aortic stenosis, aortic coarctation, hypertension) or pathological conditions such as hypertrophic cardiomyopathy (HCM), wall thickening due to myocardial infiltration (amyloidosis), or a glycogen storage disease (Fabry’s). CMR provides unique complementary information assisting the diagnostic process. The characteristic LGE findings distinguishing each entity are summarised in Table 3.

**Table 3:** Differential diagnosis of LV hypertrophy according to LGE pattern

	**HTN**	**AS**	**HCM**	**Amyloidosis**	**Anderson-Fabry**
***Pattern of hypertrophy***	Concentric	Concentric	Localised	Concentric	Concentric
***Localisation of LGE***	Any segment	Basal segments	Hypertrophied segments mainly	Circumferential	Basal inferolateral wall
***Pattern of LGE***	*Non-specific* Focal Non-subendocardial	*Non-specific* Patchy sub-endocardial or mid-wall	Patchy and/or LV-RV insertion points	Myocardial nulling difficult Diffuse sub-endocardial or sub-epicardial or Mid-wall	Patchy Mid-wall

HTN states for hypertension, AS for aortic stenosis, HCM : hypertrophic cardiomyopathy, LGE for late gadolinium enhancement.

### Secondary LV hypertrophy

Although concentric LV hypertrophy (LVH) secondary to systemic hypertension (HTN), aortic stenosis (AS), or aortic coarctation is often suspected clinically and by echocardiography, CMR assists the diagnosis and provides important prognostic information. This is of particular interest in cases with discrepancies between clinical and imaging data such as marked hypertrophy unexplained by severity and duration of HTN where another underlying associated myocardial abnormality can be detected.

In hypertensive hearts, CMR allows reproducible assessment of wall thickness and LV mass with greater accuracy compared to echocardiography which is particularly important for assessing small LV mass changes over time as a consequence of treatment but also of prognostic value as it represents an independent predictor of cardiac mortality [53,54]. Up to 50% of hypertensive patients display LGE [55,56]. Although no typical pattern of LGE has been described, focal non-subendocardial distribution predominates. While no correlation was found between presence of LGE and LVEF or LV end-diastolic dimensions, patients displaying LGE had in general greater LV mass. LGE in HTN offers new insights in risk stratification which might help to determine those patients at risk of diastolic heart failure as there is a known relationship between myocardial fibrosis and diastolic heart failure and this might have an impact upon therapeutic decision-making [57].

Similarly to hypertensive hearts, in *aortic stenosis*, LV mass predicts the development of heart failure independent of the degree of AS [58]. In addition, the severity of AS and morphology of the valve can be determined by planimetry using stacks of cine CMR perpendicular to the valve [59,60]. The severity of stenosis (peak gradient) can be quantified by velocity mapping.

LGE is a frequent finding in adaptive LVH (prevalence of 62% in AS) probably related to myocardial injury secondary to mismatch between LVH and blood supply, and depends on the severity of LV remodeling [55]. Amount of fibrosis measured by CMR correlated well with values of interstitial fibrosis obtained by histopathological analyses [61]. Whilst no specific pattern of LGE is identified in AS, LGE was generally focal and observed mainly in basal segments with a patchy distribution affecting the subendocardium or the mid-wall [55,62]. Weidemann et al confirmed that myocardial fibrosis was common in severe symptomatic AS and could be accurately quantified by CMR but appeared irreversible following surgery and related to outcome [62]. Interestingly, the incidence of LGE was not related to the severity of AS or to LVEF but rather to LV mass [55]. Recently, CMR detected mid-wall myocardial fibrosis was demonstrated as an independent predictor of mortality in patients with moderate or severe AS providing an 8-fold increase in all-cause mortality compared to similar patients without LGE [63].

However, diffuse fibrosis can be difficult to identify on standard LGE images. Myocardial signal with diffuse fibrosis will be nulled to highlight focal scar losing all information on background interstitial expansion. A growing body of publications have addressed this issue and several groups have extensively studied a way to detect interstitial fibrosis, validating T1 mapping to derive a fibrotic index [64-67] (see technical aspects’ paragraph).

### Hypertrophic Cardiomyopathy

HCM represents the most common cause of sudden cardiac death (SCD) in the young, including trained athletes, and an important substrate for heart failure disability at any age. Various genetic mutations lead to a combination of myocyte hypertrophy, disarray and fibrosis.

Although an abnormal ECG associated with marked asymmetrical septal hypertrophy and a dynamic LV outflow tract (LVOT) obstruction is almost diagnostic, individuals harbouring a genetic defect for HCM do not necessarily express clinical markers of their disease at all times during life. As a result, even normal or mildly increased LV wall thickness can be consistent with the presence of an HCM-causing mutant gene. Coexistence of HTN and HCM in a proportion of patients adds to the complexity. While HCM has been characterised by substantial LV wall thickening, Maron et al reported rather a spectrum of distribution and patterns of wall thickening [68].

CMR offers the potential for high spatial resolution images with excellent border definition allowing accurate measurement of peak wall thickness and detection of localised hypertrophy especially in the apical variant of HCM and the basal anteroseptal wall, regions more difficult to image with echocardiography. LV apical aneurysms, conferring increased risk of SCD, ventricular arrhythmias, thrombo-embolic strokes and progressive heart failure, can be identified in 2% of HCM patients using CMR [69]. In addition, the degree of LVH measured by CMR independently correlated with NT-pro-BNP values [70]. LGE, identified in up to 80% of HCM patients, represents areas of focal interstitial expansion due to myocardial fibrosis [71].

Typical patterns of LGE are patchy or confluent, generally confined to segments with greatest wall thickening and at the RV-LV insertion points [68] (Figure 2C4A).

**Figure 4:**
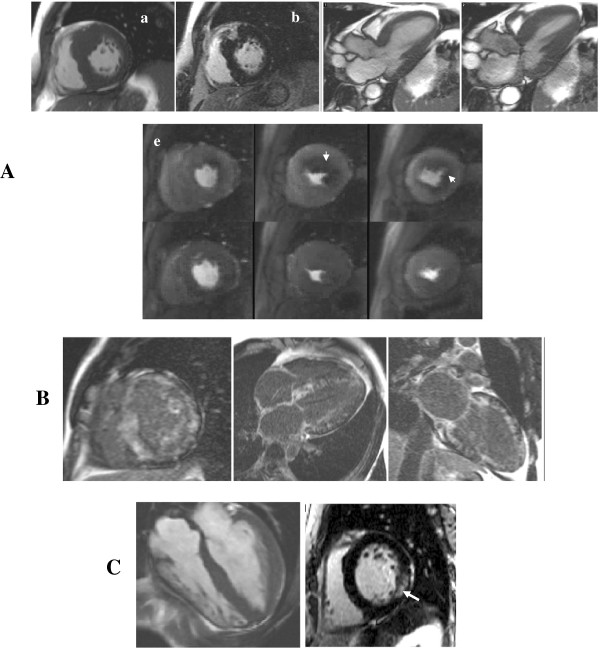
**Thickened myocardium.** (**A**) HCM with asymmetrical septal hypertrophy (**a**, arrow) and a confluent LGE pattern matching the hypertrophy (**b**, arrow). Dynamic LVOT obstruction with SAM (**c**: diastole, **d**: systole with a SAM) and circumferential sub-endocardial ischemia in hypertrophied areas as a consequence of microvascular dysfunction are among classical features (**e** top: stress-induced perfusion defects, **e** bottom: normalization at rest). (**B**) Amyloidosis with a typical circumferential diffuse enhancement (‘zebra’ pattern). (**C**) Anderson-Fabry disease with concentric LV hypertrophy (**a**) and patchy LGE affecting the inferolateral wall (**b**, arrow).

Aside from its diagnostic value, LGE extent appears related to occurrence of ventricular arrhythmias at rest and during exercise and increased risk of SCD potentially contributing to risk assessment in borderline or controversial cases benefiting from a prophylactic ICD [72,73]. Even if the incidence estimate of LGE for arrhythmic events appeared comparable to other traditional risk factors (such as hypotensive blood pressure response to exercise, gene mutations, family history of SCD, septal thickness exceeding 30 mm) available data is currently not sufficient to consider LGE as an independent risk factor for adverse prognosis [74].

CMR-detected myocardial fibrosis is associated with increased incidence of atrial fibrillation (AF) and heart failure, heralding advanced LV remodeling and systolic dysfunction [34,75]. Interestingly, while HCM patients without LGE had an excellent prognosis (100% event-free survival at 6-year follow-up), LGE involving ≥5% of LV mass, septal thickness ≥30 mm and AF were independent predictors of death and ICD discharges [76]. Microvascular dysfunction induced by coronary arteriole dysplasia or mismatch between increased LV mass and coronary flow appears as circumferential stress perfusion defects on CMR and may contribute to the risk attributable to HCM [27] (Figure 4A).

Degree of LVOT obstruction and presence of systolic anterior motion of the mitral valve can be quantified using velocity flow mapping at rest and during stress and have been correlated with more extensive hypertrophy, worse heart failure symptoms and unfavourable outcome [77]. CMR can therefore provide important clues to the differential diagnosis of secondary LVH versus HCM and offers an additional method for risk stratification.

Whilst no specific pattern of LGE has been identified in secondary LVH due to HTN or AS, focal non-subendocardial pattern has been described in the former and patchy sub-endocardial or mid-wall LGE in basal LV segments has been observed in the latter. This contrasts with the typical pattern of LGE seen in HCM patients affecting mainly insertion points and segments with maximal wall thickness (Table 3).

### Amyloidosis

This common cause of infiltrative cardiomyopathy is classified as primary, secondary (reactive), hereditary, and age-related. Myocardial involvement represents an important prognostic marker of the disease influencing treatment options [78]. Detection of early stages which may respond to therapy and exclusion of other disease mimicking amyloidosis appears crucial for patient’s management.

Typical CMR findings include a small LV cavity size with homogeneously thickened walls affecting inconsistently also the right ventricle (RV). Bi-atrial enlargement, as a consequence of the restrictive cardiomyopathy, impaired long-axis function with preserved radial function until end-stages of the disease, thickened interatrial septum and valve leaflets, pleural and pericardial effusions resulting from interstitial amyloid fibrils deposition are among the classical features. Around 55% of patients with cardiac amyloidosis may present with asymmetrical septal hypertrophy mimicking HCM.

Wassmuth et al, observed that accumulation of amyloid fibrils can change T2 relaxation times and demonstrated that myocardium appeared hypointense on T2-weighted images in cardiac amyloidosis. In addition, a lower T2 signal intensity ratio (computed as myocardial over skeletal muscle signal intensity) was independently associated with shortened survival [79]. Further evaluation is however still needed before applying it in routine practice.

While diffuse perfusion defects indicating microvascular dysfunction can be seen, the characteristic pattern of LGE and the peculiar kinetics of gadolinium are unique and discriminate this disease from other forms of hypertrophic cardiomyopathies. LGE reflects interstitial expansion by amyloid fibrils and appears as widespread, circumferential enhancement mostly of the sub-endocardium matching the distribution of amyloid protein on histology, giving rise to a characteristic ‘zebra’ pattern [78] (Figure 2B4B). This refers to the typical aspect seen in a 4-chamber view when sub-endocardial RV and LV amyloid deposition gives rise to biventricular subendocardial enhancement separated by a dark mid-wall septum. Although the most frequent finding is global sub-endocardial enhancement (up to 50% of patients), mid-wall (a third of patients) and sub-epicardial LGE (up to 20%) has also been reported [78]. Interatrial septum and RV free wall enhancement can also be associated.

Faster washout of gadolinium from blood into the total amyloid load leads to a technically challenging LGE acquisition with the inability to adequately null myocardial signal. High myocardial uptake and fast blood washout result in a dark blood pool due to similar myocardial and blood T1 values. To visualise myocardial and blood pool gadolinium kinetics before setting an inversion time when suspecting cardiac amyloidosis, an inversion time scout corresponding to an inversion-time mapping sequence with images generated at 30 ms inversion time increment, can be run early following contrast injection (less than 5 minutes). LGE has a reported sensitivity and specificity of 86% for the identification of cardiac involvement in patients with systemic amyloidosis, detecting early abnormalities in patients with normal LV wall thickness, and appeared strongly related to heart failure severity [80-82]. Additionally, gadolinium kinetics reflecting cardiac amyloid burden predicted the risk of mortality providing a novel prognostic marker [83]. Abnormal T1 intramyocardial gradient was suggested as a way to identify patients in whom early use of intensive chemotherapy might be justified and appeared as a better predictor of survival than response to chemotherapy or diastolic function in this study [83]. However, further experience with T1 intramyocardial gradient in amyloidosis and reproduction of these results are still needed.

### Anderson-Fabry disease

This treatable cause of LV hypertrophy affects 1-2% of middle aged patients suspected of HCM [84]. Concentric hypertrophy as a result of tissue deposition (lysosomal storage disease) is an early finding. However, asymmetrical septal thickening mimicking HCM can occur. CMR can suspect the diagnosis as the LGE pattern differs to that seen in HCM and displays a regional distribution which is helpful to distinguish Fabry’s disease from HCM. Almost 50% of patients have mid-wall LGE in the basal inferolateral wall related to myocardial collagen scarring specific to Fabry’s disease [85] (Figure 4C). Although the reasons for this distribution of fibrosis are unclear, this may be the substrate for electrical re-entry and SCD [86]. Regression of LV hypertrophy under enzyme replacement therapy can be accurately monitored by CMR [87]. Similarly, significant reductions in myocardial T2-relaxation time have been described witnessing the effects of enzyme replacement therapy [88]. Further validation of this method is still under way.

### Dilated cardiomyopathy (DCM)

The term DCM is applied in the presence of LV or biventricular dilatation and dysfunction following systematic exclusion of all obvious or detectable causes of cardiomyopathy. In up to 50% of cases the aetiology remains unknown, with 20-30% of familial cases attributed to known genetic abnormalities [1,89]. Myocarditis, in the chronic stage, has been described as an underlying aetiology of DCM inducing apoptosis through virus and immunocyte-mediated pathogenic mechanisms.

DCM is the second most common cause of heart failure and the most frequent cause of heart transplantation. In addition to providing accurate and reproducible assessment of ventricular volumes and LVEF, the LGE technique detects a pattern of fibrosis in DCM which is typical mid-wall and seen in one third of cases. CMR by detecting the pattern of fibrosis contributes significantly to ruling out ischaemic aetiology and providing prognostic information.

The specificity of the LGE pattern in ischaemic cardiomyopathy (ICM) has been underscored by several studies in which coronary angiography and CMR were performed in patients with systolic dysfunction of unknown aetiology [90].

**Figure 5:**
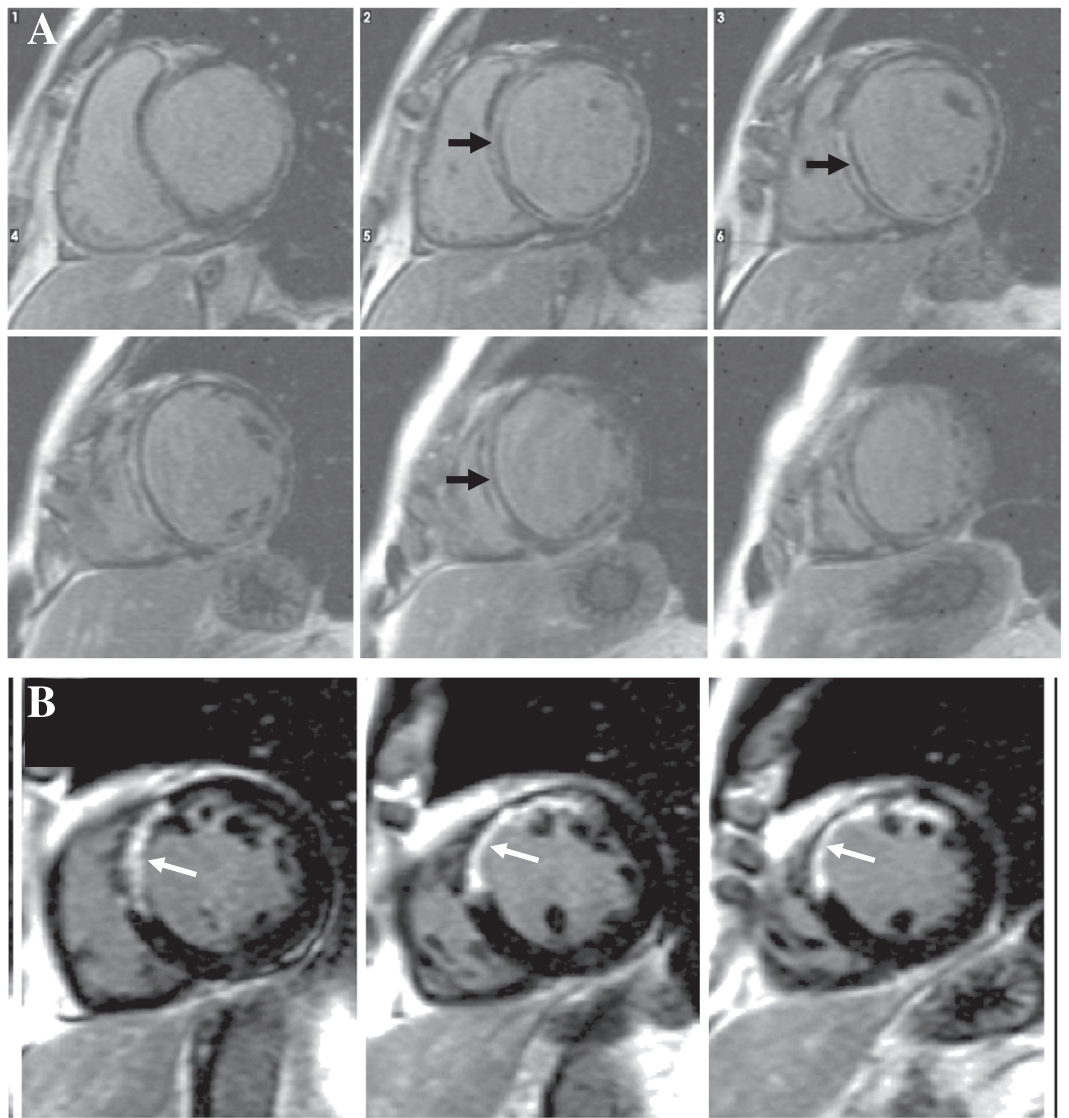
**Pattern of fibrosis in DCM.** Typical mid-wall LGE seen in DCM (**A**, arrows) differing from the sub-endocardial ischaemic pattern (**B**).

In a large study comparing patients with a diagnosis of DCM with unobstructed coronaries and patients with known coronary artery disease and a group of normal controls, three LGE patterns were reported [90]. The ischaemic pattern, with LGE spreading from the sub-endocardium to the epicardium, was seen in all patients with documented coronary artery disease. In those with unobstructed coronaries, 59% had no LGE while 28% had mid-wall LGE predominantly in the septum (Figure 5A). What was surprising was that 13% of the cohort displayed an ischaemic pattern presumably as a result of transient occlusion caused by a non-obstructive, unstable plaque or vasoconstriction (Figure 5B). Presence of an ischaemic pattern increased the risk of major cardiac events by at least sevenfold.

Presence and transmurality of fibrosis in DCM has been linked to increased risk of SCD and ventricular arrhythmias independent of other markers of risk such as reduced LVEF [91,92].

Wu et al reported an eightfold increased risk of heart failure, ventricular arrhythmias and cardiac death in DCM patients with LGE referred for an ICD [93].

Mechanisms contributing to fibrotic changes in DCM include inflammation, neurohumoral changes and microvascular ischaemia [94]. Limited CMR spatial resolution allows only the detection of areas of macroscopic replacement fibrosis responsible for promotion of re-entry mechanisms causing malignant ventricular arrhythmias.

Whilst the patterns of LGE observed in DCM patients are distinct from those seen in ICM, mid-wall but also epicardial, diffuse and focal patterns are seen [92,93]. It is still unclear whether various patterns represent different aetiologies and it is likely that a subset of patients suffered from myocarditis which developed into DCM.

Assomull et al, demonstrated recently that LGE was highly effective in detecting the underlying aetiology of newly diagnosed LV dysfunction and could be used as a safe and effective gatekeeper to coronary angiography [4]. While presence of LGE prompted further investigations with coronary angiography, absence of LGE was combined to the analysis of magnetic resonance coronary angiography to rule out major 3-vessel disease or left main stem stenosis before ascribing a diagnosis of DCM.

Pilz et al, reported previously the role of Adenosine stress CMR as a gatekeeper to coronary angiography in a different patient population demonstrating the high accuracy of CMR in detecting significant coronary artery stenoses inducing 30% reduction in the rates of coronary angiography [95].

### Arrhythmogenic right ventricular cardiomyopathy (ARVC)

A dilated RV of unexplained aetiology should raise the possibility of ARVC particularly if associated with aborted SCD from arrhythmias, often the first manifestation of the disease.

This inheritable condition predominantly involving the RV with progressive loss of myocytes and fibrofatty tissue replacement can affect the LV in up to 75% of patients. In spite of recent advances in genotyping, clinical diagnosis remains challenging.

While the classic phenotype describes progression of regional to global RV dysfunction, followed by LV involvement and biventricular failure, recognition of phenotypes with early and predominant LV involvement have raised questions regarding its differentiation from other myocardial disorders, such as DCM. Diagnosis relies largely on the ARVC Taskforce criteria, recently revised to include quantitative parameters for imaging studies, improving sensitivity of detection of gene-carriers with limited disease expression and those with left-sided disease features [96].

These published cut-off values for RVEF and end-diastolic volumes (Major criteria : Regional wall motion abnormality and RV end-diastolic volume ≥110 ml/m^2^ for male and ≥100 ml/m^2^ for female or RVEF ≤40%; Minor criteria : Regional wall motion abnormality and RV end-diastolic volume ≥100- <110 ml/m^2^ for male and ≥90- < 100 ml/m^2^ for female or RVEF >40% - ≤45%) have been defined on the basis of comparison with normal patient data.

Luijkx et al applied these modified criteria to help distinguish ARVC from physiological adaptation seen in athletes. RVEF and LV/RV end-diastolic volume ratio appeared as the most discriminant parameters in this situation [97].

CMR is therefore an integral component of the clinical evaluation and represents the gold standard for assessing the RV when performed in dedicated centers by experienced operators, enhancing the sensitivity of diagnosis particularly in early phases of the disease.

**Figure 6:**
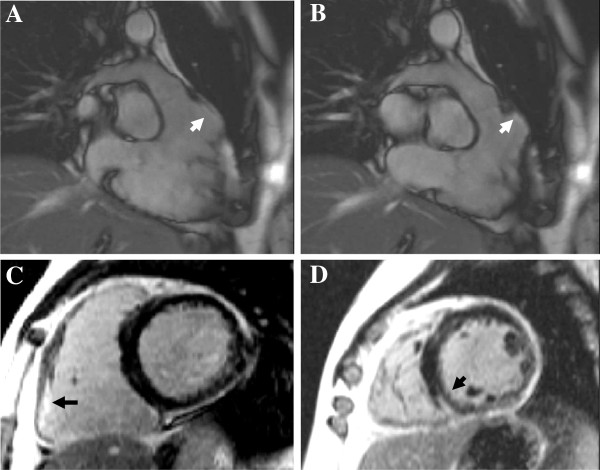
**ARVC.** Findings suggestive of ARVC include localized aneurysms in the RV outflow tract (arrows, **A**: diastole, **B**: systole), LGE of the free wall (**C**, arrow) and in the left-sided form areas of mid-wall LGE at the RV-LV insertion point (**D**, arrow).

Regional and global RV function, wall motion abnormalities, aneurysms or areas of thinning are best detected by series of high temporal resolution trans-axial cine of the RV reaching 96% sensitivity and 78% specificity when part of a comprehensive non-invasive work-up (Figure 6) [98].

T1-weighted spin echo images used initially to image myocardial fat proved of limited diagnostic value since adipose infiltration of the RV occurs also in healthy individuals and is difficult to image in the thin RV wall.

LGE as part of a dedicated protocol appears as the most discriminating factor with a high diagnostic sensitivity and specificity [98]. While RV LGE is difficult to distinguish from myocardial fat and requires substantially different inversion times compared to the LV, LV mid-wall or sub-epicardial LGE can contribute to delineate different patterns of ARVC. In the classic form, mid-wall LGE is seen in the inferolateral and inferior wall while the left-dominant phenotype often mid-diagnosed as DCM, may include prominent LV mid-wall enhancement affecting the septum with preserved RV function [99].

Although the prognostic value of CMR is still under investigation, LGE appears to predict inducibility of sustained ventricular tachycardia and occurrence of RV dysfunction [100]. However, lack of specificity of LGE pattern combined with revision of taskforce imaging criteria (requiring the combination of wall motion abnormality with global RV dilatation or dysfunction) may not have improved the sensitivity of detection of ARVC despite keeping high specificity [101]. This highlights the importance of a combined diagnostic approach (clinical, electrophysiological and imaging).

### Chest pain syndromes with unobstructed coronaries

CMR provides unique diagnostic information in patients with cardiomyopathy of unknown aetiology presenting with troponin positive chest pain syndromes (CPS) unexplained by significant coronary lesions [102]. In a cohort of 130 patients, CMR contributed to reach a diagnosis in 77% of patients presenting with CPS with unobstructed coronaries, correcting a wrong suspected diagnosis in 10% of patients and leading to change in therapy in 33% [103].

**Figure 7:**
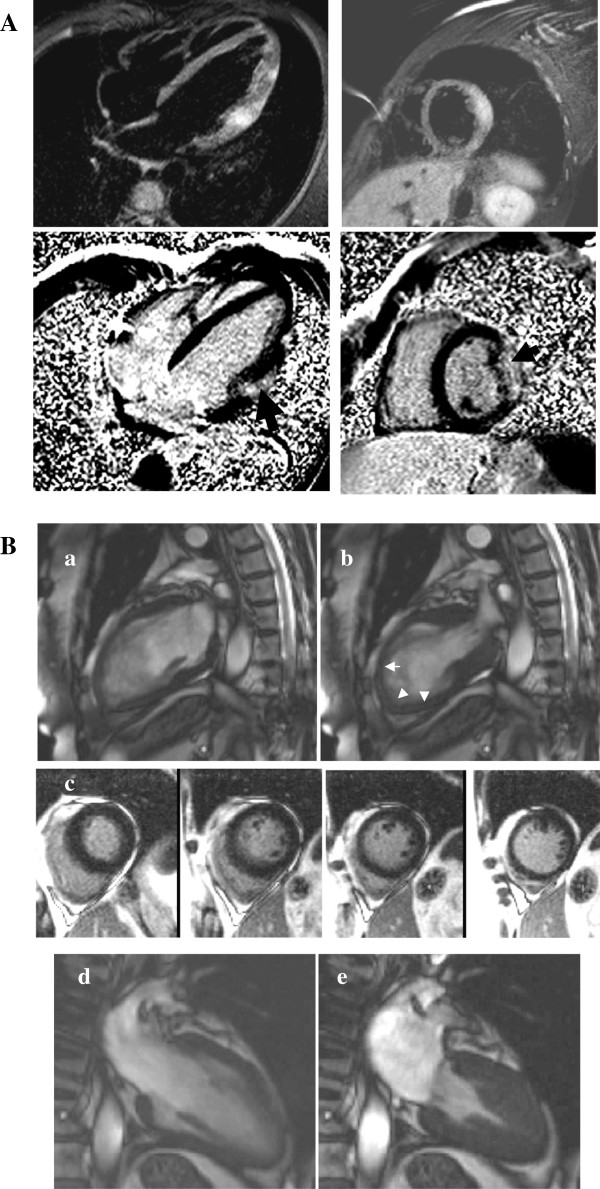
**Acute chest pain syndromes (A) Acute viral myocarditis displaying edema on STIR images (a-b, arrows) and typical sub-epicardial LGE (c-d, arrows).** (**B**) Tako-tsubo with acute apical ballooning of the LV (**a**: diastole, **b**: systole displaying apical akinesia, arrows) without LGE (**c**) followed by complete LV functional recovery at follow-up (**d**: diastole, **e**: systole).

Key features rely on the use of T2-weighted imaging increasing the sensitivity and specificity of detection of an ischaemic insult and on the typical pattern of LGE distinguishing an ICM from an NICMP and detection of myocarditis and Tako-tsubo [104] (Figure 5B and 7).

### Myocarditis

Myocarditis occurs with a wide spectrum of clinical manifestations ranging from flu-like symptoms to troponin positive CPS, severe heart failure and SCD. Endomyocardial biopsy recognised as the gold standard for the diagnosis remains limited by insensitivity and sampling bias.

CMR can image several aspects of myocardial injury.

In the acute stage of the disease, oedema following lymphocytic infiltration and myocytolysis appears as regions of high intensity on T2-weighted images. EGE matching areas of T2-hyperintensity may reflects tissue hyperemia and increased interstitial space [105].

LGE identifies inflammation and fibrosis acutely and areas of irreversible necrosis in later stages, typically affecting the sub-epicardium sparing the sub-endocardium of the lateral wall (Figure 7A). Mid-wall LGE has also been described in myocarditis [106]. This distribution pattern seems specific for viral myocarditis with a relationship between affected segment and type of virus [106]. The localisation of myocardial damage can guide endomyocardial biopsy enhancing diagnostic accuracy. Pericardial hyperintensity on T2-weighted images associated to pericardial effusion detect associated pericarditis.

Among patients presenting with troponin positive CPS with unobstructed coronaries, CMR was able to identify a cause in 65% of patients with the commonest being myocarditis [107]. In patients with unexplained cardiomyopathy, up to 10% of patients were found to have CMR findings of myocarditis [17]. Beside its diagnostic value, increased LGE at 4 weeks after clinical onset has been inversely correlated with 3-year follow-up LVEF [108].

A large consensus group provided recommendations on the use of CMR in patients with suspected myocarditis suggesting the combination of 3 CMR criteria (‘Lake Louise criteria’) to increase the diagnostic accuracy [18]. Authors recommend the combined use of 3 tissue markers : regional or global hypersignal intensity on T2-weighted images, increased EGE (ratio between myocardium and skeletal muscle) and at least 1 focal lesion on LGE with a non-ischaemic distribution. When 2 or more of these tissue-based criteria were positive, myocarditis could be ruled out or predicted with a diagnostic accuracy of 78% as compared with endomyocardial biopsy. However, diagnostic accuracy of CMR in various clinical, histological and immunohistochemical subgroups still needs large multicenter trials validation [18].

### Tako-tsubo cardiomyopathy

Acute but rapidly reversible LV systolic dysfunction in absence of coronary artery disease, triggered by profound psychological stress characterises this stress-mediated myocardial stunning. Mid and apical LV segments akinesia with ballooning and compensatory hyperkinesia of basal segments producing a dynamic obstruction is a typical finding. RV is involved in 25% of patients.

Focal and transient transmural hyperintensity on T2-weighted imaging reflecting oedema matching the distribution of wall motion abnormality seems to be a feature of Tako-tsubo cardiomyopathy related to the severity of LV systolic dysfunction [19]. Lack of perfusion defect or LGE is typical, contrary to myocarditis and infarction [109] (Figure 7B). However, up to 33% of patients display mid-wall patchy LGE at 24 hours with complete resolution at follow-up corresponding to reactive transient gadolinium accumulation related to increased wall stress [110,111]. In contrast to other cardiomyopathies such as myocarditis, presence of LGE did not correlate with worse outcome. Complete recovery of LV function at follow-up is typical.

Recently, Eitel et al analysed the CMR findings in Tako-tsubo cardiomyopathy in a large multicenter prospective cohort [112]. These observations highlighted the presence of a much broader clinical profile than previously reported with a significant percentage of men and younger and only two-thirds of patients exposed to a preceding stressor. CMR demonstrated a variety of ballooning patterns with the apical one being the most frequent, followed by mid-ventricular (17%), biventricular and basal (1%) types. Authors identified several diagnostic criteria requiring further validation in other cohorts combining severe LV dysfunction in a non-coronary regional distribution pattern, myocardial oedema concordant to the regional wall motion abnormality (verified by a quantitative signal intensity analysis), absence of LGE, increased EGE and complete resolution after 4 weeks.

### CMR features of other cardiomyopathies

#### Sarcoidosis

This multisystem granulomatous disease of unknown aetiology involves the myocardium in 20-30% of cases.

Clinical myocardial involvement (5% of patients) ranges from asymptomatic conduction abnormalities to heart failure or fatal ventricular arrhythmias. An early diagnosis is crucial as current therapy may effectively prevent death from arrhythmias and cardiac failure. Endomyocardial biopsy and echocardiography proved insensitive diagnostic tools detecting mostly advanced stage of the disease.

**Figure 8:**
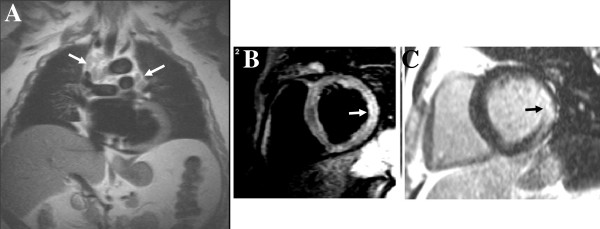
**Sarcoidosis.** Hilar lymphadenopathy on HASTE (**A**, arrows), localized edema in the lateral wall on STIR images (**B**, arrow) and mid-wall LGE (**C**, arrow) are suggestive of sarcoidosis.

Although results of prospective data are awaited, CMR appears promising for the early diagnosis and for monitoring treatment response, providing novel risk stratification criteria for SCD and heart failure. T2-weighted and STIR imaging detect localised areas of myocardial inflammation. Cines identify areas of thinning and regional wall motion abnormalities and LGE detects focal scarring, typically patchy, mid-wall or sub-epicardial, frequently involving the anteroseptal and anterolateral walls [113] (Figure 8).

Patel et al described a large spectrum of LGE patterns. While the majority of affected myocardial segments had non-transmural involvement, explaining normal ventricular wall motion, there was a predilection for basal and mid-ventricular septum with up to 48% of regions displaying sub-endocardial LGE similar to coronary artery disease LGE. However, 86% of affected patients had also at least one region of LGE with a non-ischaemic type pattern. Sub-endocardial LGE of the RV side of the septum was a common finding and could be associated with LGE of the RV free wall [114].

CMR appeared more sensitive for disease detection compared to Thallium SPECT and gallium SPECT [115]. Recent studies demonstrated that 18 F-2-fluoro-2-deoxyglucose positron emission tomography (FDG-PET) was as sensitive as CMR for detection of earlier stages of sarcoidosis [116]. A structured clinical assessment incorporating imaging with FDG-PET and CMR appeared more sensitive than the established clinical criteria. Using the widely described Japanese Ministry of Health and Welfare criteria as a gold standard, CMR had a diagnostic sensitivity of 100% [117]. LGE appeared as twice as sensitive for detection of cardiac sarcoidosis as current consensus criteria and portended a ninefold higher rate of adverse events including cardiac death [114]. Patients with LGE had a higher rate of diastolic dysfunction, reduced RVEF and evidence of non-sustained ventricular tachycardia [118]. Interestingly, LGE may guide localisation for endomyocardial biopsy and monitor the efficacy of steroid therapy [119]. However, LGE pattern is non-specific and the predictive value of a negative CMR remains unknown.

### LV non-compaction (LVNC)

This rare congenital disorder is characterised by a “spongy” appearance of the LV (and the RV in <50% of cases) resulting from an arrest in normal embryogenesis.

LVNC may be associated with neuromuscular disorders, leading to heart failure, arrhythmias and thrombo-embolic manifestations. Although echocardiography is considered as the reference for the diagnosis, CMR accurately diagnosed LVNC delineating hyper-trabeculations of the apex and the lateral wall [120].

**Figure 9:**
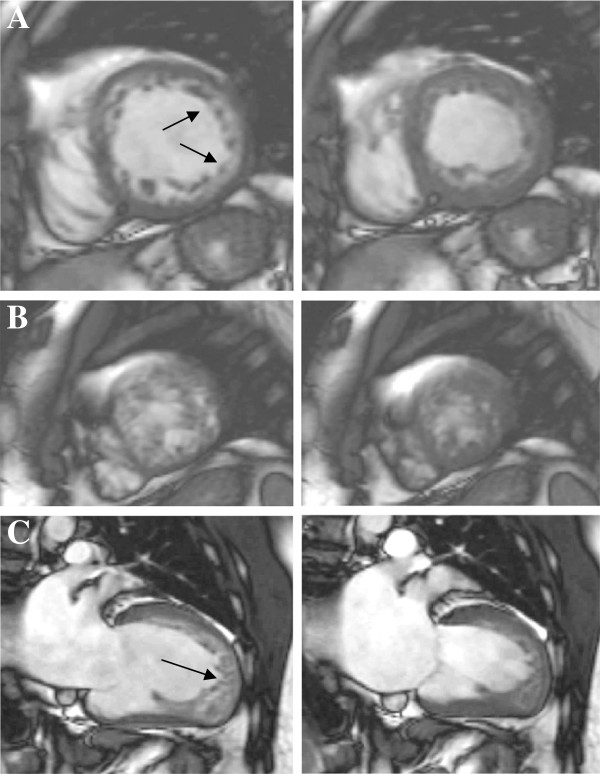
Left ventricular noncompaction LVNC with marked LV apical and lateral wall trabeculations, arrows (left panel: diastole, right panel: systole, A: mid-segment, B: apical segment, C: 2-chamber view).

Hyper-trabeculation with a end-diastolic ratio of non-compacted over compacted myocardium of 2.3 distinguished pathological from non-pathological conditions with a sensitivity of 86% and specificity of 99% [120] (Figure 9). Although a variety of morphological findings are described, the absence of well-formed papillary muscles is a clue to the diagnosis. Trabecular sub-endocardial, mid-wall or transmural LGE and sub-endocardial perfusion defects suggestive of areas of microvascular dysfunction have been reported. Dodd et al noticed a correlation between extent of LVNC, amount of trabecular LGE and LVEF (35). Similarly, Nucifora et al, suggested that presence and extent of LGE were independently related to LVEF [121]. However, the prognostic value of these findings has yet to be shown.

### Iron-overload cardiomyopathy

Heart failure due to myocardial iron overload remains the leading cause of death in patients with transfusion-dependant anemias. Reversibility of this condition relies on early and intensive iron chelation. CMR has made possible the accurate measurements of both liver and cardiac iron in the same study detecting myocardial involvement.

**Figure 10:**
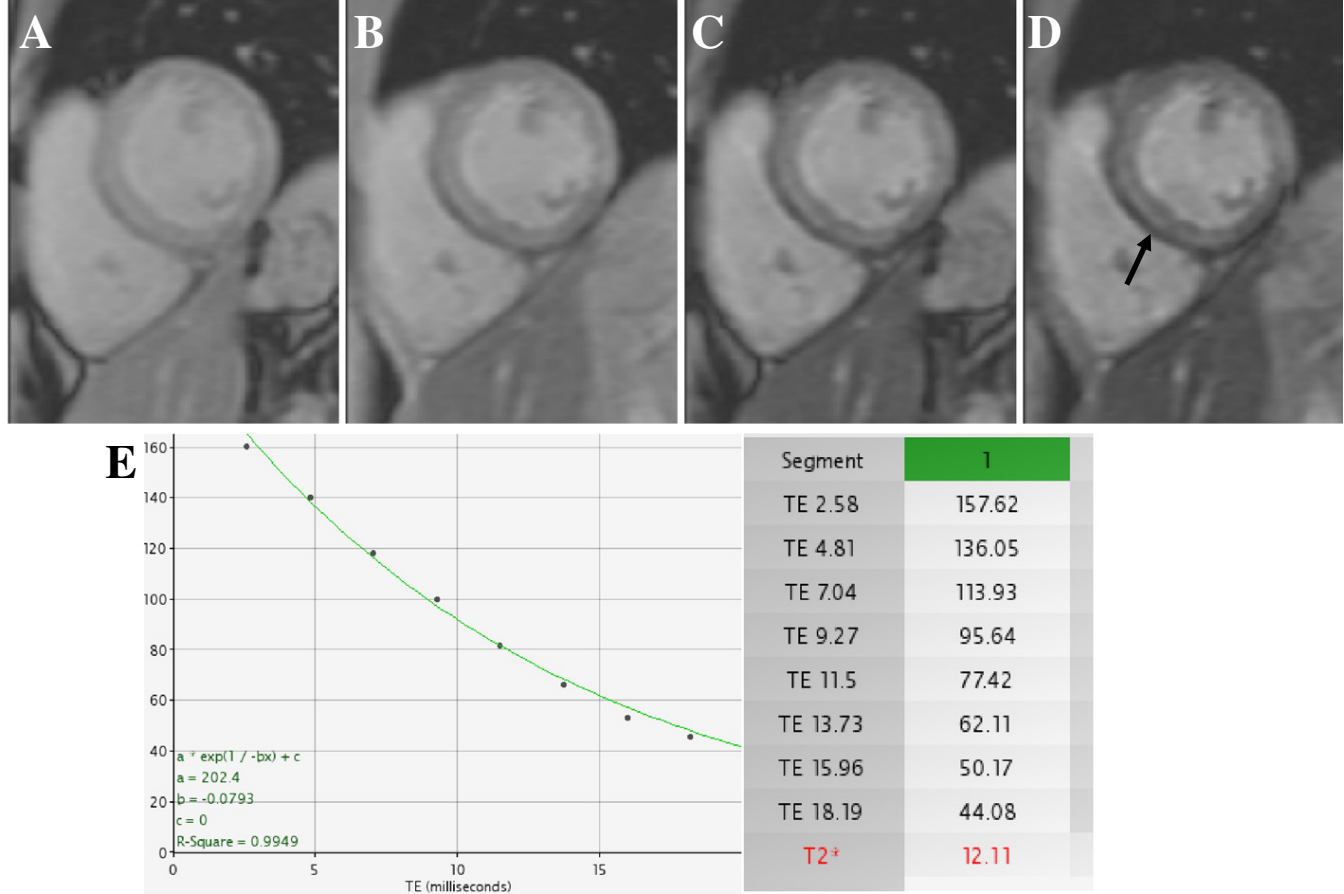
**Myocardial iron overload.** A mid-level gradient echo short-axis slice is imaged at different echo times (**A**: 2 ms to **D**: 14 ms) and T2*value is estimated from the signal intensity decay curve (**E**). Typical sub-epicardial iron deposition can be seen (arrow).

Anderson et al, first reported the utility of T2* imaging to detect and quantify myocardial iron, unrelated to the extent of liver iron deposition [25]. Myocardial T2* arises principally from local magnetic field inhomogeneities that are increased with greater iron deposition. Typical sub-epicardial iron deposition in mid-septum is visualised by acquiring a mid short-axis slice at nine separate echo times to derive the T2* value (Figure 10). Reduced T2* relaxation times <10 ms found in thalassemia patients with new onset heart failure defines severe cardiac iron overload and strongly relates to decreased LVEF. T2* has been used as a predictive marker of heart failure and arrhythmias but also to direct treatment and monitor chelation therapy’s efficiency, resulting in 80% reduction in mortality rates for this condition in the United Kingdom [122].

Carpenter et al, confirmed recently that mid-ventricular septal iron, measured ex-vivo in human hearts from thalassemia patients, and using the CMR relaxation parameter R2* (assessed clinically as its reciprocal, T2*) were highly representative of mean global myocardial iron validating current clinical practice [123].

Interestingly, macroscopic fibrosis detected by LGE is uncommon in thalassaemia patients despite current or prior heart failure and across a broad spectrum of myocardial iron loading [124].

### Transplant cardiomyopathy

Despite significant advances in anti-rejection therapy, acute cellular allograft rejection remains a leading cause of early mortality among transplanted patients. The diagnostic gold standard technique remains repeated endomyocardial biopsy largely limited by insensitivity.

CMR can assist the detection of rejection mainly through myocardial T2 relaxation time quantification with sensitivities and specificities approaching 90% except for the early peri-operative period characterised by normal post-operative oedema [125]. A complete normalisation of T2 relaxation times was seen following treatment of rejection. Marie et al, suggested that CMR sensitivity might help to predict rejection in some biopsy-negative patients [126]. Beyond the first year, transplant coronary artery disease (TCAD) is the second most common cause of death after malignancy. Patterns and prevalence of LGE were investigated in heart transplant patients undergoing routine coronary angiography [127]. While 50% had a non-ischaemic pattern of LGE, 30% of event-free patients with unobstructed coronaries had a typical infarction pattern associated with worse LV function and higher BNP values. LGE might therefore help earlier diagnosis of TCAD allowing intensification of therapy in selected patients. Data on prognostic implications are awaited.

### Chagas disease

Cardiac involvement by Trypanosoma cruzi infection occurs in 30% of seropositive patients representing a major cause of death from heart failure in Latin America.

LGE-CMR typically involves the apical and mid-inferolateral wall in patients with advanced disease but also in asymptomatic seropositive subjects without wall motion abnormalities [128]. Ischaemic and non-ischaemic LGE patterns are reported evolving toward areas of thinning, aneurysms and LV dysfunction. Myocardial fibrosis correlated inversely with LVEF and clinical status. Preliminary data suggest a direct relationship between amount of fibrosis and patient’s prognosis [129].

### Chemotherapy induced-cardiomyopathy

The most common adverse effect of chemotherapy is cardiotoxicity affecting patient’s prognosis and quality of life independently of the oncological issue. A wide range of agents have been associated with cardiotoxicity, anthracyclines and related compounds being the most frequently implicated.[130]. Incidence of clinical heart failure varies from 1 to 5% and asymptomatic LV dysfunction ranges from 5 to 20%. Toxicity can occur early (within a year) or be delayed. Two types of cardiac toxicity have been described: type 1 resulting in permanent damages to the myocardium due to cell death and type 2 due to cellular dysfunction following drug toxicity and usually reversible. Cumulative dose, age at the time of drug exposure, concomitant use of other chemotherapeutic agents, chest irradiation and preexisting cardiovascular disease are among risk factors to develop chemotherapy-induced cardiomyopathy.

While echocardiography remains the commonest imaging modality used to follow-up LVEF in patients at risk of developing cardiotoxicity, case series assessing the role of CMR in this setting have raised interesting issues. In a population of patients receiving trastuzumab therapy for breast cancer and developing LV dysfunction, authors reported the presence of sub-epicardial LGE in the lateral wall in all patients as a sign of myocarditis induced by drug toxicity [131,132]. Other authors suggested that absence of LGE was associated with reversibility of LV dysfunction [133]. However, these preliminary findings still need confirmatory studies by other groups.

### Differential diagnosis of NICMP-Practical tips

An optimal nulling of the myocardium is key to LGE interpretation and detection of small areas of fibrosis or quantification of sub-endocardial scars. Although the optimal inversion time can be set based on experience, a single short axis slice can be acquired automatically at different inversion times. The optimal inversion time is then selected visually as the one nulling completely the healthy myocardial signal. According to the underlying suspected pathology, LGE should be acquired before 10 minutes such as in amyloidosis due to the faster washout of gadolinium from blood pool. In cardiomyopathy patients, gadolinium washout can also be faster, needing an earlier acquisition to obtain optimal nulling of the myocardium. This is thought to be related to structural alterations of extracellular matrix associated with reduced myocardial blood flow in DCM patients [39]. Once the optimal inversion time is chosen, it should be adjusted throughout the scan, typically increased from 310 to 420 ms. Presence of a subtle area of LGE is confirmed if myocardial enhancement persists when reimaged in the same plane with a different orientation phase (phase swap) ruling out an artifact. Similarly, performing crosscuts through the suspected region of LGE (performing another plane cutting the area in question) will assure presence of fibrosis and help define its transmurality within the wall. Similar acquisition techniques can be used for STIR imaging.

### When to order a CMR scan

Lack of ionising radiation, excellent spatial resolution, 3D anatomical assessment and ability to provide tissue characterisation with and without contrast are amongst advantages of CMR making it a valuable and complementary tool to other non-invasive modalities. CMR is recognised as a class I indication for EF, volumes and mass quantification [9]. CMR should therefore be performed to quantify EF in patients with poor echocardiographic window and in controversial cases where precise determination of EF will impact upon decision regarding treatment, device implantation or follow-up (i.e ICD or CRT). In these specific cases, CMR can contribute to the decision-making process by adding important prognostic information reflected by LGE burden as reviewed in previous sections. In addition, CMR represents the most reproducible tool for LV mass measurements at baseline and during follow-up under treatment. CMR should also be an adjunct to the work-up of all patients with suspected cardiomyopathy due to its ability to study the RV, to detect subtle wall motion abnormalities or reduction in LVEF, to identify myocardial oedema and small areas of fibrosis or scarring.

LGE CMR appears highly effective in identifying the aetiology of LV dysfunction in newly diagnosed heart failure and can be used as a safe, clinically effective and potentially economical gatekeeper to coronary angiography in these patients [4]. Safety of repeated imaging allows screening of cardiomyopathy family members or athletes who experienced unexplained syncope or aborted SCD. CPS with unobstructed coronaries represent another condition where CMR has the unique capability of discriminating infarction from myocarditis or tako-tsubo cardiomyopathy driving the clinical management of these patients. To promote the quality and impact of CMR imaging and to be able to set international benchmarks on appropriate indications, quality and safety of CMR, a European registry, EuroCMR registry is currently recruiting patients’ data from a large number of participating centers [134].

### Comparison with other Imaging modalities

Whilst CMR offers an excellent morphological evaluation through its high spatial resolution, echocardiography excels through its unique temporal resolution, providing a non-ionising, inexpensive bedside tool, superior for haemodynamic evaluation (filling pressures, diastolic function, restrictive physiology, valve disease). Limitations are linked to its restricted lateral spatial resolution, the dependency on good acoustic windows particularly in patients with chronic lung diseases, and the limited assessment of apical segments and the RV. CMR remains partially limited by arrhythmias interfering with ECG triggering and poor ability to perform breath-holds. Metallic non-CMR safe devices and severe renal failure prevent its use.

Cardiac computed-tomography (CCT) provides a fast and accurate 3D assessment of coronaries and myocardium in a 30-second time frame even in patients with pacing devices. However, the use of CT remains limited by radiation, renal failure often encountered in heart failure patients (for CT contrast agents), pregnancy and limited assessment of late enhancement still inferior to CMR. It can however be considered as an alternative in patients with pacing devices.

Nuclear and CT hybrid devices are rapidly evolving to incorporate high-speed MDCT along with positron-emission tomography (PET) and single-photon emission tomography (SPECT) detector systems.

This dual modality imaging presents an opportunity to use a single piece of equipment for a combined assessment of coronary anatomy, perfusion, function and metabolism or coronary calcification. However, radiation exposure as well as availability and cost remain an issue. Table 4 summarises the diagnostic ability of various imaging techniques extensively used in the work-up of a newly diagnosed cardiomyopathy. Table 5 summarises the main CMR findings of most frequent NICMP.

**Table 4:** Comparison of Imaging Modalities for the evaluation of NICM

	**Echo**	**CMR**	**CT**	**SPECT**	**PET**
**Scan time**	15-25 min	30-45 min	10 min	2 hours	1 h^1/ 2^-2 hours
**Radiation**	None	None	1.5-2 mSv (64-slice Coronary CT)	41 mSv (Thallium stress/rest) 9 mSv (Sestamibi)	14 mSv (F-18 FDG)
			6–10 mSv (multi-detector row CT)		
			1–1.3 mSv		
			1.5-6 mSv (multi-detector coronary calcium scoring) [124]		
**Risks**	None	NSF (related to some types of gadolinium-based contrast if severe renal failure)	Radiation Renal failure Allergy	Radiation Allergy (rare)	Radiation Allergy (rare)
**Contra-indications**	None	MRI-incompatible implants and devices Pregnancy during first trimester (precautionary)	Renal failure Pregnancy	Pregnancy	Pregnancy
**Limitations**	Operator dependent Acoustic window (obese, COPD) Imaging of apical segments and RV (spatial resolution)	Availability Lower temporal resolution	Not ideal for serial follow-up owing to radiation Currently not suited for detection of fibrosis, perfusion and wall motion Blood flow cannot be assessed	Availability Low spatial resolution	Availability Low spatial resolution
**Accuracy of LV /RV function and volumes**	++ 3D echo	+++	-	++	-
**Wall thickness /Mass quantification**	+ (localised hypertrophy can be missed)	+++	+	-	-
**Detection of oedema**	- (non-specific findings such as wall thickening)	+++ (STIR sequences)	-	+ (non-specific; areas of reduced perfusion)	+++ FDG (uptake)
**Imaging of fibrosis**	- (suspected with 2D strain)	+++	+	-	+
**Detection of microvascular disease/ Stress Perfusion**	+ Contrast echo Stress echo	+++	+ Perfusion	++ [125]	+++ (considered gold standard)
**Assessment of Myocyte Metabolism**	-	+ (Field of research, CMR spectroscopy)	-	+ (technical limitations, quantitative methods unavailable)	+++

**Table 5:** Summary of the main CMR findings in various NICMP

	**Cine**	**STIR**	**Stress Perfusion**	**EGE**	**LGE**
**HCM**	Localised area of hypertrophy LV apical aneurysms	Normal	Circumferential defects (microvascular dysfunction)	Normal or hyposignal intensity in the area of perfusion defect	Patchy or confluent in regions of maximal wall thickening and LV/RV insertion points
**Amyloidosis**	Homogeneously thickened LV wall Thickened RV wall, interatrial septum, valves Pericardial effusion	Myocardial hypointensity	Diffuse perfusion defect	Diffuse perfusion defect	Widespread circumferential (commonest) Mid-wall Sub-epicardial Inter-atrial septum RV wall
**Anderson-Fabry**	Concentric hypertrophy	Normal	Not assessed with CMR	Normal	Mid-wall in basal inferolateral wall
					Sub-epicardial (rarely)
**DCM**	Dilated LV and/or RV Reduced LVEF	Normal	Normal	Normal or hyposignal intensity within the LV if thrombus	Mid-wall septal (commonest) Epicardial Diffuse
**ARVC**	Dilated RV				
	RV dysfunction	-	-	RV free wall (difficult to image)	
	Focal aneurysms				Mid-wall septal, inferolateral or inferior
	Local hypo or akinesia				
					LV wall (ALVC)
**Myocarditis**	Wall motion abnormalities	Focal epicardial or transmural hypersignal intensity	Not performed in the acute setting	Global EGE	Focal sub-epicardial
	Localised thickened myocardium				
	LV dysfunction				
	Pericardial effusion				
**Tako-tsubo**	Mid and apical akinesia (ballooning)	Focal, transient transmural	Normal	Normal	Normal
	Compensatory basal hyperkinesia	hyper-intensity (apical segments)			
**Sarcoidosis**	Normal or	Focal areas of hypersignal intensity	-	Focal	Focal patchy, mid-wall or sub-epicardial or subendocardial
	Wall motion abnormalities or thinning				Basal and mid-septum mostly
	LV and/or RV dysfunction				Sub-endocardium of RV side of the septum and RV free wall
**LVNC**	Hyper-trabeculation (non-compacted over compacted > 2.3)	Normal	Sub-endocardial perfusion defects	Sub-endocardial lack of EGE	Sub-endocardial, mid-wall or transmural trabecular enhancement
	Absence of well-formed papillary muscles				

### Future directions

CMR spectroscopy (MRS) and CMR-based molecular imaging have appeared recently as non-invasive tools for assessing myocardial metabolism using the intrinsic properties of nuclei including Phosporus-31, proton, sodium-23, oxygen-17 and 13-carbon [135]. Phosphorus-31 is the most widely used form of spectroscopy and allows detection of adenosine -5’ triphosphate (ATP) and phosphocreatine (PCr), the high energy phosphate compounds of the heart. This allows identification and quantification of deranged cardiac energy metabolism in heart failure [136]. Thus, PCr: ATP ratios appeared reduced in heart failure correlating with functional class and LVEF, but were also reported as strong predictors of prognosis and long-term survival [137]. Recently, absolute quantification of PCr and ATP was achieved demonstrating a marked reduction of these elements in heart failure associated with a reduced rate of turnover of ATP, a sensitive parameter of metabolic derangement. MRS holds promising perspectives to monitor energetic changes linked to conventional or novel heart failure treatments which improved PCr:ATP ratios together with clinical improvement [138]. Recently, Proton MRS has been used to quantify myocardial triglyceride content as an early marker of diabetic heart disease [139]. MRS offers new insights in the assessment of prognosis and monitoring of therapeutic strategies in heart failure but remains limited by low temporal and spatial resolution and low reproducibility. Additionally, available studies are still limited by their small sample size.

Newly developed quantitative Oxygen-sensitive CMR techniques have been designed to directly evaluate myocardial oxygen consumption [140]. Elevated deoxyhemoglobin seen in a territory subtented by a stenotic coronary artery can be assessed by blood oxygen level dependent (BOLD) CMR. BOLD CMR relies on paramagnetic properties of deoxyhemoglobin leading to signal loss in T2 and T2*-weighted sequences thus acting as a natural contrast agent [141-145]. This method provides new insights in the evaluation of myocardial blood flow (MBF), myocardial blood volume (MBV), fractional oxygen extraction (OEF) and myocardial oxygen consumption (MVO_2_)[143]. MBF measured during stress showed the best correlation with PET data in a canine model of severe coronary artery stenosis [143]. Interestingly, Karamitsos et al showed that regional myocardial perfusion and oxygenation were dissociated in a significant proportion of segments in patients with coronary artery disease demonstrating that reduced perfusion does not always lead to deoxygenation whereas normal perfusion is usually associated with normal oxygenation. Friedrich et al found a significant correlation between BOLD T2* signal intensity changes and thallium-SPECT in patients with coronary artery disease [145]. These growing techniques offer potential possible alternatives to nuclear techniques and new insights into pathophysiology of diseases.

## Conclusions

Through a wide range of sequences, CMR offers a unique non-invasive tool which should be integral part of the clinical work-up of a cardiomyopathy.

In vivo tissue characterisation remains a major strength of CMR compared to other non-invasive techniques detecting and distinguishing oedema from fat, fibrosis and myocardial infiltration. Its excellent spatial resolution set it above other imaging techniques to analyse RV mophology and detect RV cardiomyopathies and for identification of regional ventricular hypertrophy or dilatation.

Although, CMR provides precious and irreplaceable additional information to standard imaging and invasive methods, it cannot be seen as an alternative to these techniques yet and as a one stop shop but as a unique supplementary method able to characterise abnormal tissue in vivo, allowing pre-clinical detection of disease process and novel parameters for risk stratification.

## Competing interests

The authors declare that they have no competing interests.

## Authors’ contributions

All authors contributed to the design and writing of this manuscript. All authors read and approved the final manuscript.
